# Die Kosten der Niedrigzinspolitik am Wohnungsmarkt. Immobilienpreisblasen in B-Lagen am Beispiel Salzburg

**DOI:** 10.1007/s00548-022-00791-5

**Published:** 2022-07-05

**Authors:** Andreas Van-Hametner

**Affiliations:** Paris Lodron Universität Salzburg, Salzburg, Österreich

**Keywords:** Wohnungsmarkt, Nullzinsen, Betongold, COVID-19, Immobilienblase, Housing market, Zero interest, “Concrete gold”, COVID-19, Real-estate bubble

## Abstract

In großen Teilen Europas, so auch in Deutschland und Österreich, boomt die Immobiliennachfrage. Immer häufiger wird Wohnraum aber nicht zum Wohnen, sondern als Anlage erworben. Neben institutionellen InvestorInnen kaufen zunehmend auch private AnlegerInnen, vor allem auf exponierten Märkten, die bislang schon einen Nachfrageüberhang aufwiesen. Wesentlich dazu beigetragen hat die Nullzinspolitik der EZB. Der Boom führt in vielen Städten zu Immobilienpreisblasen. Um das gestiegene Blasenrisiko abseits der Metropolen aufzeigen zu können, verwende ich am Beispiel der Stadt Salzburg ein Set aus vier Indikatoren: Die Leistbarkeit von Wohneigentum, die Rentabilität von Vermietungen, die Entwicklung der Fertigstellungen von Wohneinheiten sowie die Entwicklung der Wohnungsbaukredite. Mit diesen Indikatoren können regionale PlanerInnen und Politik Marktentwicklungen einschätzen und Preisblasen identifizieren. Sie zeigen für das Beispiel Salzburg eine deutliche regionale Überhitzung, welche auch durch die COVID-19-Pandemie nicht gestoppt wurde. Im Gegenteil haben die davon beeinflussten Nachfragemuster den aktuellen Boom sogar noch beflügelt. Beton ist mehr denn je „Gold“!

## Abstract (en)

Demand for real estate is booming in large parts of Europe, including Germany and Austria. Increasingly, it is not purchased to live in, but as an investment. In addition to institutional investors, private investors are increasingly buying too, especially in exposed markets that are already showing excess demand. The zero interest rate policy of the European Central Bank contributed significantly to this. The boom is leading to property price bubbles in many cities. To be able to show the increased risk of price bubbles outside the metropolises, I use a set of four indicators with the example of Salzburg: the affordability of home ownership, the profitability of rentals, the growth of completed residential units, and the development of housing construction loans. With these indicators, regional planners and politicians can assess market developments and identify price bubbles. For the example of Salzburg, they show clear regional overheating, which was not even stopped by the COVID-19 pandemic. On the contrary, the demand patterns influenced by it have actually stimulated the current boom. More than ever, concrete is “gold”!

## Boomende Wohnungsmärkte in Deutschland und Österreich

In ganz Europa stehen die Wohnungsmärkte unter Druck wie lange nicht. Die COVID-19-Pandemie hat die boomende Nachfrage sogar noch befeuert. Alleine in den fünf größten europäischen Immobilienstandorten London, die Metropolregionen Amsterdam und Berlin, Paris und Wien wurden 2020 bei großen Immobiliendeals (mindestens 10 Wohnungen) über 16 Mrd. € investiert. Von 2007–2020 lag dieser Wert in Berlin gar bei 40 Mrd. €, in Wien bei 11 Mrd. € (vgl. Tagesspiegel Innovation Lab 2021).

Diese Dynamik ist aber nicht auf große Ballungsräume beschränkt. Deutschlandweit wuchs der gewerbliche Wohninvestmentmarkt zwischen 2010 und 2020 von 3,3 auf 21,7 Mrd. € (JLL 2021). Auch der österreichische Wohnimmobilienmarkt stieg deutlich an, 2019 wurden um 43 % mehr Eigentumswohnungen verkauft als 2010 und selbst im ersten COVID-Jahr 2020 legte der Markt leicht zu (RE/MAX 2020). Die Wohnungsmärkte von Deutschland und Österreich weisen aber nicht nur aktuell ähnliche Dynamiken auf, sondern haben auch vergleichbare Strukturen: Beiden gemein sind im OECD-Vergleich niedrige Wohneigentumsquoten, regulierte Mietmärkte und aufgrund vergleichbarem Wohlstandsniveau ähnliche Nachfragestrukturen. Auf der Angebotsseite weist Österreich noch stärker soziale Wohnungsbestände auf wie Deutschland, da dort bereits mehr Bestände privatisiert wurden (Voigtländer 2007).

Warum in diesen beiden Ländern Investitionen in Wohnimmobilien derart boomen, welche Rolle Betongold sowie die Nullzinspolitik der EZB dabei spielen und ob diese Entwicklung auch abseits der Metropolen zu Preisblasen führt, analysiert dieser Beitrag anhand des Beispiels der Stadt Salzburg. Zudem skizziert er eine Methodik, welche die Planungspraxis dabei unterstützt, regionale Marktanalysen durchzuführen, die Marktentwicklung korrekt einzuschätzen und Preisblasen zu identifizieren. Denn das Erkennen von Preisblasen ist nicht nur aus wissenschaftlicher Perspektive interessant. Die Analyse und Bewertung von Preisentwicklungen ist ebenso für PlanerInnen relevant. Im Besonderen für die planerische und stadtpolitische Reaktion auf sich verschärfende Wohnkrisen ist die eingehende Analyse der Begründungszusammenhänge von Immobilien- und Preisboom unabdingbar. Bislang kann aber in vielen Städten nicht auf regional differenzierte Untersuchungen zurückgegriffen und so regional auftretenden Überhitzungen nur unzureichend erkannt und begegnet werden. Das vorliegende Beispiel Salzburg zeigt also exemplarisch eine Analysemöglichkeit für weitere deutsche und österreichische Städte vergleichbarer Größe.

### Die Nullzinspolitik und Wohnraum als Kapitalanlage

**Abb. 1:**
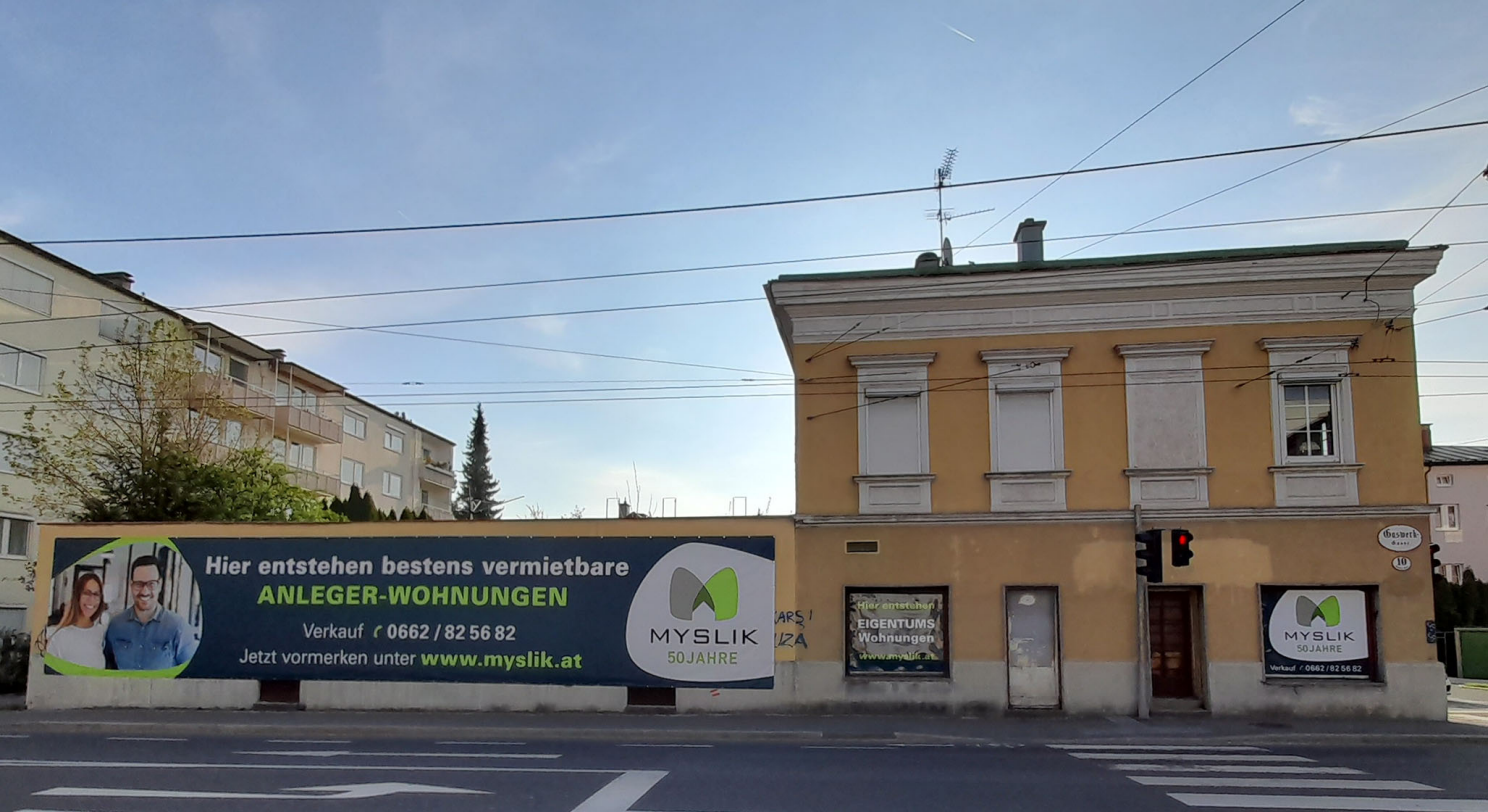
Werbung für Anlegerwohnungen in Salzburg. (Foto: Autor)

Eine wesentliche Rolle für den derzeitigen Immobilienboom spielt die Nachfrage nach Wohnraum als Kapitalanlage. Zwar wurden die Immobilienmärkte bereits seit den 1980er-Jahren für Finanzinvestments geöffnet und Wohnraum zu einer standardisierten Ware (Heeg 2004). Doch traten anfänglich vor allem institutionelle InvestorInnen am Wohnungsmarkt auf. Seit der Finanzkrise 2008 kaufen aber zunehmend auch Privatpersonen Wohnungen zur Anlage, da sie von finanzmarktbasierten Anlageformen abgeschreckt sind. Sie kaufen Immobilien sowohl indirekt (z. B. durch Immobilienfonds) als auch direkt durch private Immobilieninvestitionen in Form von Anlage- bzw. Vorsorgewohnungen. Das sind vorwiegend kleinere Eigentumswohnungen, die mit oder ohne Vermietungsabsicht zur Vorsorge oder als Finanzanlage erworben werden (EHL 2020; Van-Hametner 2021). Sowohl in Deutschland wie auch in Österreich kam es in den letzten Jahren zu einer deutlichen Zunahme dieser Käufe durch Privatpersonen (EHL 2020; Hüttig und Rompf 2021) (Abb. 1).

**Abb. 2:**
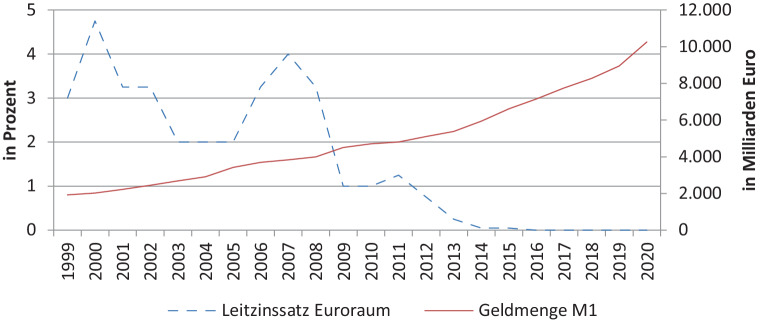
Europäische Geld- und Zinspolitik. (Quelle: OeNB 2021b; EZB 2021)

Wesentlich dazu beigetragen hat einerseits die Niedrig- bzw. seit 2016 Nullzinspolitik der EZB, da sie konservative Sparformen unattraktiver machte. Andererseits führte die gleichzeitige Erhöhung der Geldmenge (in Abb. 2 eng gefasst als M1 = Bargeld und Sichteinlagen) durch die EZB und ihre Anleihekaufprogramme – gedacht zur Konjunkturbelebung und Deflationsvorbeugung – zu einer „Geldschwemme“ und zu Inflationssorgen. Beides trug dazu bei, dass private AnlegerInnen ihr Kapital noch stärker in Sachvermögen wie Immobilien investieren. Sie erwarten von ihrer Investition in Wohnraum (auch wegen ihrer Unterschätzung der Mietausfallsquote) trotz niedriger Renditen Schutz vor der gefürchteten Inflation.

### Überhitzung in und abseits der Ballungsräume

Mehrere Untersuchungen attestieren Deutschland wie Österreich aufgrund des aktuellen Booms Überhitzungstendenzen. Bereits 2018 sah die deutsche Bundesbank Überbewertungen in deutschen Großstädten von bis zu 30 %. Lange Zeit konzentrierte sich das deutlichste Blasenrisiko auf die großen Ballungsräume – in Deutschland die 12 größten Städte (empirica 2021a), in Österreich Wien (OeNB 2021a). Mittlerweile geht der empirica Blasenindex (2021a) aber von einer deutschlandweiten Preisblase aus, und auch in Österreich warnt die Nationalbank vor einer bundesweiten Überbewertung von 19 % (OeNB 2021a). Neben den Ballungszentren sind zusehends auch kleinere Großstädte, Mittelstädte sowie das metropolitane Umfeld von dieser Dynamik betroffen, die dort vor allem von lokalen Privatanlegern getrieben wird (Van-Hametner 2021; F+B 2021).

### COVID-19 und der Wohnungsmarkt

Die Entwicklungen im Zuge der COVID-19-Pandemie beschleunigten die Preisanstiege von Wohnungseigentum in diesen Klein- und Mittelstädten (F+B 2021) und verstärkten die Gefahr von Überhitzungen. Zwar sind noch nicht alle Implikationen eindeutig, doch zeichnen sich folgende Auswirkungen ab:

- *Angebot und Nachfrage: *Der zu Beginn der Pandemie entstandene Rückgang an Fertigstellungen wurde bereits Ende 2020 mehr als wettgemacht. Arbeitslosigkeit und Kurzarbeit ließen die Einkommen vieler Haushalte sinken und reduzierten die Möglichkeit, Eigentum zu erwerben. Aus den Erfahrungen der Lockdowns vollzog sich eine Rückbesinnung auf den privaten Raum und eine Änderung der Nachfragemuster. Gefragt ist mehr denn je Wohnen im Grünen, Platz für die gestiegenen Bedürfnisse nach Homeoffice-Infrastruktur zu Hause sowie Wohnraum zur Anlage (Raiffeisen RESEARCH 2020).
- *Preise: *Historische Vergleiche zeigen, dass Pandemien temporär zu einem deutlichen Rückgang der Immobilienpreise führen können. In Deutschland stieg allerdings der empirica-Immobilienindex (2021b) vom ersten Quartal 2020 auf das zweite Quartal 2021 um über 11 % und auch in Österreich verteuerten sich Immobilien im gleichen Zeitraum um 17 % (OeNB 2021a).

## Preisblasen erkennen

Wie können boomende nun von überhitzten Märkten unterschieden werden und welche Konsequenzen hat diese Einschätzung? Eine Methode dafür besteht im Abgleich von Preisentwicklung und fundamentalen Angebots- und Nachfragefaktoren (u. a. demografische Entwicklung, allgemeines Wohlstandsniveau, gesellschaftliche Präferenzen oder erwartbare Erträge (Schneider 2014)) in ökonometrischen Modellen. Weichen diese deutlich voneinander ab, so liegt eine Unter- oder Überbewertung vor. Dies ist zum Beispiel dann der Fall, wenn die Nachfrage nach Wohneigentum nicht durch das natürliche Wohnbedürfnis, sondern von Spekulation und übertriebener Erwartung auf zukünftige Wertsteigerungen angetrieben wird. Dies kann dazu führen, dass Immobilienkäufer Preise bezahlen, die weder durch ihr Einkommen noch durch zu erwartende Renditen gerechtfertigt sind. Methodisch besteht abseits der Metropolen dabei das Problem oftmals fehlender Daten. Um das Blasenrisiko aber auch abseits der Ballungsräume einzuschätzen, kann ein einfaches Indikatorenset aus folgenden vier Parametern (vgl. empirica 2021a) verwendet werden:

1. *Leistbarkeit von Wohneigentum* (Price-to-income-Ratio): Dabei wird das durchschnittliche Einkommen in einer Region in Bezug zu den regionalen Immobilienpreisen gesetzt und die Frage gestellt, wie (un)erschwinglich Wohneigentum für EigennutzerInnen ist.
2. *Rentabilität von Vermietungen* (Price-to-rent-Ratio): Das Verhältnis von Immobilienpreisen zu Mietpreisen gibt einerseits an, welcher Immobilien-Teilmarkt gerade attraktiver ist (bzw. ob es sinnvoller ist, als EigennutzerIn eine Wohnung zu kaufen oder zu mieten) und andererseits, welche Mietrendite möglich ist (bzw. wie gut Neukäufe mit zu erwartenden Mieteinnahmen refinanzierbar sind).
3. *Fertigstellungen zu Bevölkerungsentwicklung*: Dieser Parameter lässt erkennen, ob die Überbewertung mit einem (spekulativen) Bauboom einhergeht, also viele Wohnungen errichtet werden, ohne dass dies durch die Nachfrage von EigennutzerInnen gedeckt ist.
4. *Wohnbaukredite zu Haushaltseinkommen*: Nehmen diese deutlich zu, bedeutet dies, dass Wohneigentum immer stärker fremdkapitalfinanziert erworben wird.

Sinken die Leistbarkeit der EigennutzerInnen und die Rentabilität von Vermietungen und steigen gleichzeitig die Fertigstellungen und Wohnungsbaukredite an, so ist eine Immobilienpreisblase naheliegend (empirica 2021a). Denn wenn weder für EigennutzerInnen noch AnlegerInnen der Immobilienerwerb ökonomisch-rational begründbar ist, kann deren Handeln zumeist nur durch spekulative Investitionsmotive erklärt werden. Auch die aktuell vorherrschende Investition in Betongold als Inflationsschutz kann als spekulatives Investitionsmotiv betrachtet werden, liegen doch auch ihr keine Fundamentalwerte zugrunde. Führt dies zu verstärkten Neubau, um die spekulative Nachfrage zu befriedigen und steigen damit auch immer mehr KäuferInnen ein, die auf hohe Fremdkapitalanteile angewiesen sind, weitet sich die volkswirtschaftliche Gefahr aus.

## Preisblasen auf dem Immobilienmarkt der Stadt Salzburg

Um das gestiegene Blasenrisiko abseits der Metropolen aufzuzeigen, verwende ich nun am Beispiel der Stadt Salzburg dieses Set aus vier Indikatoren. Salzburg ist eine Landeshauptstadt und mit knapp 150.000 BürgerInnen, eine der teuersten und dynamischsten Immobilienmärkte Österreichs. Neubauwohnungen kosteten 2020 pro m^2^ 5796 €, Bestandswohnungen 4149 € (Hölzl und Hubner 2021) und der freie Nettomietzins lag bei 10,24 €/m^2^ (WKO 2021). Dies bedeutet eine deutliche Zunahme seit 2008, vor allem der Eigentumspreise im Neubau (+79 %) und Bestand (+132 %), die durch die COVID-19-Pandemie nur verlangsamt wurde.

### Leistbarkeit von Wohneigentum

**Abb. 3:**
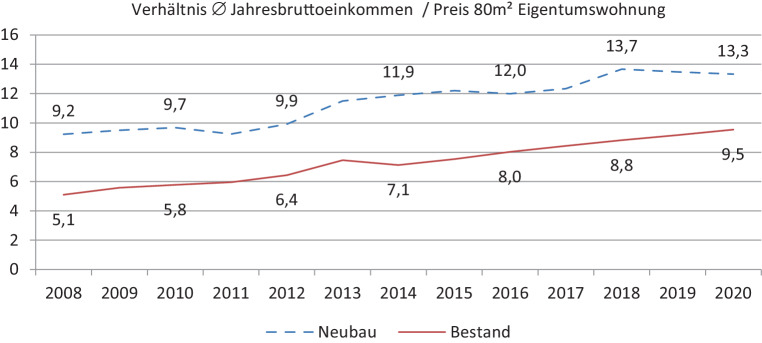
Price-to-income-Ratio in Salzburg 2008–2020. (Quelle: AK Salzburg (2021); Hölzl und Hubner (2021); eigene Berechnungen)

Mit dieser Entwicklung der Immobilienpreise konnten die Einkommen der Salzburger Haushalte (AK Salzburg 2021) nicht Schritt halten. Zwar stiegen sie in den letzten 12 Jahren kontinuierlich, aber geringer als die Immobilienpreise. So sank die Leistbarkeit von Wohneigentum deutlich. Musste ein Haushalt für eine 80 m^2^ Neubauwohnung 2008 im Durchschnitt noch etwas mehr als 9 Jahreseinkommen (brutto) ausgeben, lag dieser Wert 2020 bei 13,3. Für Bestandswohnungen mussten statt 5,1 sogar 9,5 Jahreseinkommen aufgewendet werden. Immer weniger SalzburgerInnen können sich deshalb Eigentum leisten (Abb. 3).

### Rentabilität von Vermietungen

**Abb. 4:**
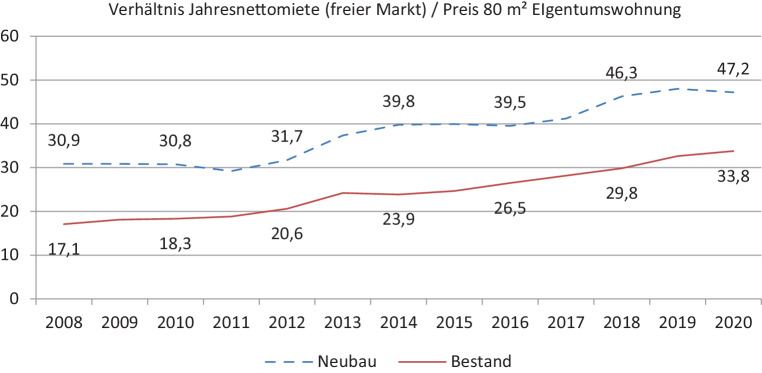
Price-to-rent-Ratio in Salzburg 2008–2020. (Quelle: Hölzl und Hubner (2021); WKO (2021); eigene Berechnungen)

So wie die Einkommen, konnten auch die Mietpreise (trotz Steigerungen über der Inflation) mit der Dynamik der Eigentumspreise nicht Schritt halten. Die Price-to-rent-Ratio (Abb. 4) zeigt eine zurückgehende Rentabilität von Vermietungen in Salzburg. Als renditeorientierte Anlage wird Wohneigentum deshalb zunehmend weniger interessant. Auch im ersten Jahr der COVID-19-Pandemie setzte sich diese Entwicklung für Bestandsimmobilien fort. Im Neubau reduzierte sich die Price-to-rent-Ratio minimal, liegt aber nach wie vor auf dem Niveau von A‑Lagen (vgl. empirica 2021a).

### Fertigstellungen zu Bevölkerungsentwicklung

**Abb. 5:**
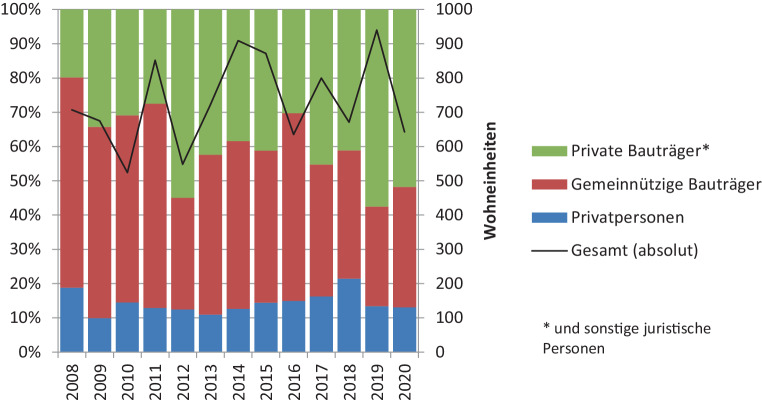
Wohnungszugang nach Bauherr. (Quelle: Stadt Salzburg (2008–2020); eigene Darstellung)

Doch geht mit den stark gestiegenen Immobilienpreisen auch ein Bauboom in Salzburg einher und falls ja, was wird prioritär errichtet? Die Zeitspanne seit der Krise 2008 ist von keiner deutlichen Zunahme der Fertigstellungen gekennzeichnet. Die Daten zeigen einen typischen regionalen Immobilienmarkt, dessen Output in Abhängigkeit größerer Einzelprojekte variiert. Auch der Rückgang im Jahr 2020 ist deutlich, aber nicht außergewöhnlich. Geändert hat sich hingegen, wie in weiten Teilen Deutschlands und Österreichs, die Zusammensetzung des Neubaus. Der freifinanzierte Wohnbau boomt, zulasten des gemeinnützigen Wohnbaus (vgl. Zeller et al. 2018). Zudem werden mehr Eigentums- wie Mietwohnungen errichtet. Die Zunahme an neu errichteten Wohnungen geht in Salzburg aus unterschiedlichen Gründen (u. a. Leerstand durch Betongold, Zunahme Einpersonenhaushalte) nicht mit einer Bevölkerungszunahme einher (Abb. 5).

### Wohnbaukredite zu Haushaltseinkommen

**Abb. 6:**
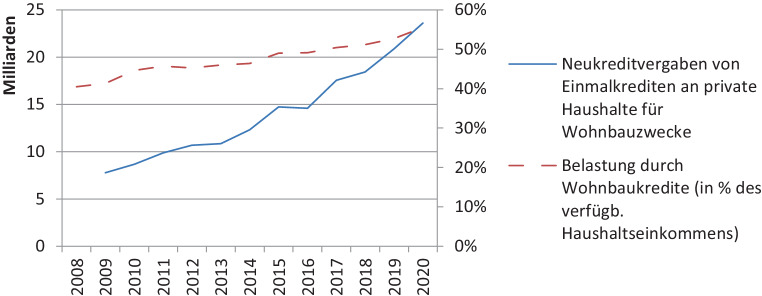
Entwicklung der Neuvergabe und Belastung durch Wohnbaukredite in Österreich. (Quelle: OeNB (2021a, c); eigene Darstellung)

Zur Finanzierung von Wohnimmobilien gibt es keine regionalisierten Statistiken. Daten für ganz Österreich zeigen, dass die Belastung durch Wohnbaukredite in den letzten Jahren zunahm und auch in den ersten Monaten der COVID-19-Pandemie nochmals zulegte. Die Belastung durch Zinsausgaben blieb hingegen gleich. Andererseits kam es in den letzten Jahren auch zu einem Boom der Neukreditvergaben an private Haushalte für Wohnbauzwecke. Immobilienkäufe sind also immer höher – zwar festverzinst, aber – fremdfinanziert (Abb. 6).

## Fazit

In großen Teilen Europas, so auch in Deutschland und Österreich boomt, angetrieben durch Anlagen institutioneller und privater InvestorInnen, die Nachfrage nach Immobilien. Wesentlich dazu beigetragen haben die aktuelle europäische Zins- und Geldpolitik. Auch die COVID-19-Pandemie hat diese Entwicklung nicht gestoppt, sondern sogar noch beflügelt.

**Abb. 7:**
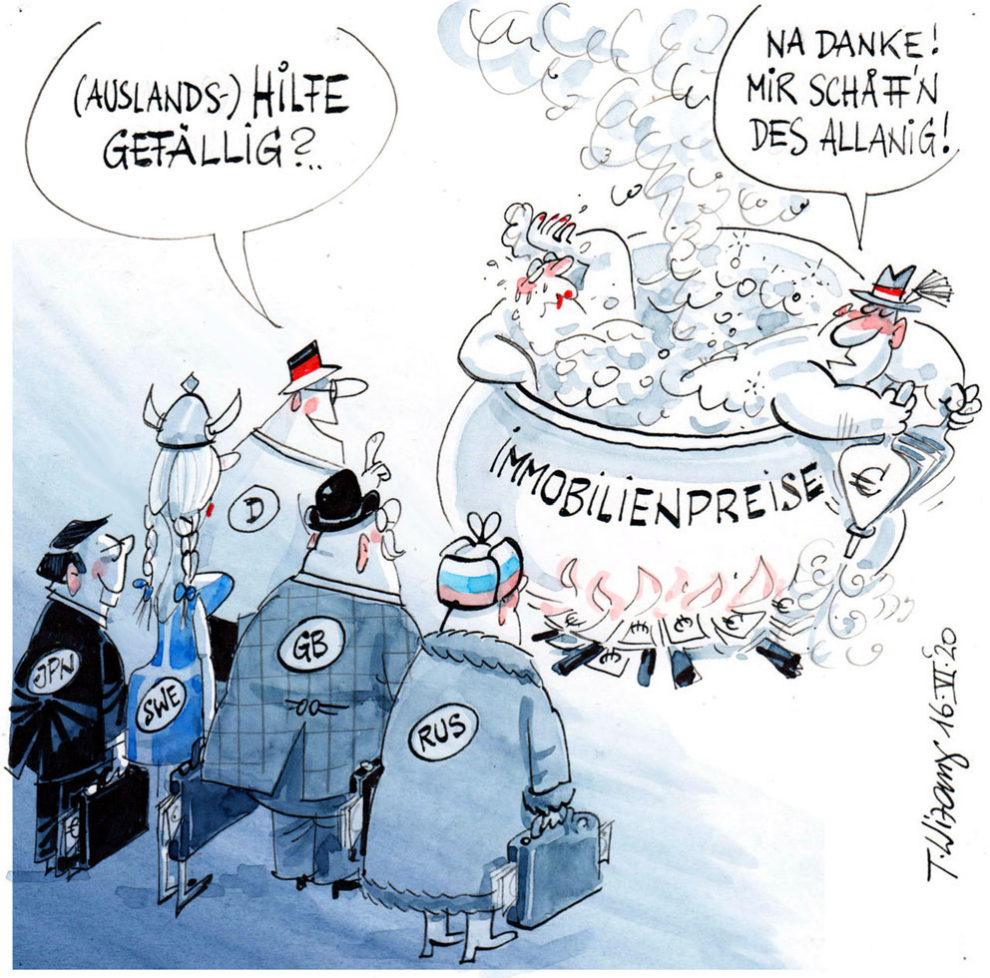
Privatanleger kommen zumeist aus der Region. (Foto: Wizany/Salzburger Nachrichten)

Der Boom führt in vielen Städten zu Immobilienpreisblasen. Um das gestiegene Blasenrisiko abseits der Metropolen zu analysieren, beurteilt dieser Beitrag die Entwicklungen am Beispiel der Stadt Salzburg stellvertretend für andere boomende deutsche und österreichische Städte. Sowohl die finanzielle Leistungsfähigkeit der Haushalte als auch die Rentabilität von Vermietungen sind in Salzburg gesunken. Trotzdem bleibt die Nachfrage nach Wohneigentum im Besonderen von PrivatanlegerInnen (in Form von Anlage- und Vorsorgewohnungen), aber auch EigennutzerInnen hoch. Nach den ersten Schüben der COVID-19-Pandemie traten als Erstes wieder AnlegerInnen in ihrer Suche nach einem Ausweg aus der Nullzinssituation am Markt auf (vgl. Salzburger Nachrichten 11.05.2020; 09.09.2021). Da aber sowohl Leistbarkeit wie Rentabilität gesunken sind, kaufen beide Gruppen zu ungerechtfertigt hohen Preisen. Die deutlichen Preissteigerungen sind somit auf eine Überbewertung zurückzuführen. Demgegenüber haben die Fertigstellungen von Wohneinheiten durch den Preisboom nicht zugenommen. Außerdem ist diese Überbewertung „hausgemacht“, denn es kaufen vorwiegend KleininvestorInnen aus der Region (karikiert dargestellt in Abb. 7), bei denen nicht Wertsteigerung, sondern die Sicherheit der Veranlagung im Vordergrund steht (Van-Hametner 2021). Auf sie richtet sich die Wohnungswirtschaft zunehmend aus. Zwar nahmen Neukreditvergaben in den letzten Jahren deutlich zu, doch sind diese in zunehmendem Maße festverzinst. AnlagekäuferInnen hingegen finanzieren eher mit privaten Ersparnissen und brauchen oft keine Kredite (Van-Hametner 2021). Aus diesen Gründen führt die Überbewertung zu keiner Marktinstabilität, ein plötzlicher Preissturz durch Panikverkäufe ist nicht zu erwarten.

Was kann die regionale Planungspraxis nun gegen die Überhitzung eines regionalen Wohnungsmarkts tun? Zentral ist, die Marktentwicklung richtig einschätzen und Preisblasen identifizieren zu können. Zwar können sie die europäische Zinspolitik nicht regional ändern, aber dazu beitragen, die Angriffsfläche für Anlagekapital gering zu halten. Je mehr Eigentumsimmobilien auf einem Standort errichtet werden, desto mehr Anlagemöglichkeiten bieten sich. Je höher der Anteil des freien Wohnungsmarkts ist, desto geringer sind die Einflussmöglichkeiten der öffentlichen Hand dämpfend einzuwirken und desto höher sind die Wohnkosten (Gutheil-Knopp-Kirchwald 2021). Das Entstehen von Preisblasen, beeinflusst durch Anlageinvestitionen im Zuge der aktuellen Niedrigzinspolitik ist eine große Herausforderung – aber kein Naturgesetz!
